# Inequities in HIV/AIDS mortality in 315 Latin American cities: a population-based cross-sectional analysis

**DOI:** 10.1016/j.lana.2026.101580

**Published:** 2026-07-20

**Authors:** Sebastián Genero, Andrés Trotta, Laio Magno, Mónica Serena Perner, Tania Alfaro, Diana Higuera-Mendieta, Usama Bilal, Marcio Alazraqui

**Affiliations:** a Instituto de Salud Colectiva, Universidad Nacional de Lanús, Lanús, Argentina; b Instituto Gonçalo Moniz, Fundação Oswaldo Cruz, Salvador, Bahia, Brazil; c Departamento de Ciências da Vida, Universidade do Estado da Bahia, Salvador, Bahia, Brazil; d Universidad Nacional de Río Negro, Bariloche, Argentina; e Escuela de Salud Pública, Facultad de Medicina, Universidad de Chile, Santiago, Chile; f Urban Health Collaborative, Dornsife School of Public Health, Drexel University, Philadelphia, PA, USA; g Department of Epidemiology and Biostatistics, Dornsife School of Public Health, Drexel University, Philadelphia, PA, USA

**Keywords:** HIV/AIDS mortality, Health inequities, Social inequalities, Urban health, Latin America

## Abstract

**Background:**

HIV/AIDS mortality trends to concentrate in large urban settings but studies on HIV/AIDS mortality in Latin American cities remain scarce. This study aimed to describe HIV/AIDS mortality and its association with indicators of deprivation and income inequality in 315 Latin American cities.

**Methods:**

This was a cross-sectional ecological study of 315 cities from eight Latin American countries, with cities as the unit of analysis. We calculated city-level HIV/AIDS-related mortality rates from 2016 to 2020 data for all countries, except Chile, for which 2015–2019 data were available, using empirical Bayes models. We estimated the proportion of the variability in HIV/AIDS-related mortality that was between-country and between-city. Associations with city socio-environmental conditions and city income inequality were examined, overall and by sex, through multilevel regression models.

**Findings:**

Mortality rates were higher among men than women across all countries. Between-city differences explained 48·3% of the variance in men and 41·5% in women. After adjustment for country and age, each one-point increase in the city-level Social Environment Index (SEI) z-score was associated with a 25% (95% CI 15–30%) lower risk of death among women and a 19% (95% CI 11–26%) lower risk among men. This association varied by age and sex, with the strongest effects observed in those aged 14 years or younger.

**Interpretation:**

HIV/AIDS mortality in the cities examined shows marked inequalities by sex, age, and place, with a substantial proportion of variability explained by urban-level conditions. Better socio-environmental conditions were associated with reduced mortality, underscoring the importance of addressing structural determinants to reduce urban health inequities.

**Funding:**

The Salud Urbana en América Latina (SALURBAL)–Urban Health in Latin America project is funded by the Wellcome Trust.

**Research in context**

**Evidence before this study**

We searched PubMed on December 30, 2024, to identify studies published in English, Spanish, or Portuguese analyzing inequalities in HIV/AIDS mortality in the general population across multiple urban settings in different countries. We used the following strategy: (HIV [Title/Abstract] OR AIDS [Title/Abstract]) AND (mortality [Title/Abstract]) AND (city [Title/Abstract] OR cities [Title/Abstract] OR urban [Title/Abstract]) AND (inequality [Title/Abstract] OR disparity [Title/Abstract] OR inequity [Title/Abstract]). We also searched OpenAlex and SciELO, adapting these terms to the specific search strategies of each database. SciELO was included to ensure the identification of studies published in Latin American journals.

Of the 30 studies initially identified, only one met the inclusion criteria. This ecological study examined inequalities in HIV/AIDS mortality across municipalities in six Latin American countries — Brazil, Colombia, Costa Rica, Ecuador, Guatemala, and Mexico — over the period 2000–2017. In the final year of the study period, age-standardized HIV/AIDS mortality rates differed by more than 40-fold among men and 20-fold among women across municipalities at the extremes of the distribution. Mortality was concentrated in high-population municipalities, and a shift toward older age groups was observed over the study period.

**Added value of this study**

To our knowledge, this is the first study to examine the distribution and association between absolute and relative deprivation and HIV/AIDS-related mortality by age and sex using harmonized and comparable data from large cities in Argentina, Brazil, Colombia, Guatemala, Panama, El Salvador, and Mexico during the period 2016–2020 and Chile 2015–2019. HIV/AIDS-related mortality was lower in magnitude but more unequally distributed among women, particularly in the 50–69 age group. A substantial variability in HIV/AIDS-related mortality was attributable to between-city differences. Cities with lower absolute deprivation had lower HIV/AIDS-related mortality rates across all age and sex groups, with the exception of women aged 70 or older.

**Implications of all the available evidence**

Taken together, the findings indicate that HIV/AIDS-related mortality in Latin America -largely preventable with timely and effective interventions-varies across cities according to levels of absolute deprivation. Our analysis illustrates how disaggregated, city-level data can reveal inequalities and HIV/AIDS-related mortality patterns in that would otherwise not be apparent using more aggregated approaches. These results underscore the need for geographically targeted and equity-oriented HIV prevention and care strategies incorporate age- and sex-specific approaches, and address broader structural determinants of health. Strengthening subnational surveillance systems and integrating social deprivation indicators into public health planning could enhance the effectiveness of interventions aimed at reducing avoidable HIV/AIDS-related mortality and health inequalities in the region.

## Introduction

Globally, HIV/AIDS-related mortality has been declining since 2006, but there is substantial heterogeneity in population levels and temporal trends between and within countries.^1^ Many HIV/AIDS-related deaths are preventable, as guaranteeing access to a continuum of healthcare can allow people living with HIV to achieve a life expectancy comparable to that of the general population.^2^ This is reflected in the ambitious global goal of reaching zero AIDS-related deaths by 2030.^3^

Despite this progress, major inequalities in mortality due HIV/AIDS persist, particularly among the most vulnerable populations due to social, structural, and political barriers.^4^^,^^5^ For instance, although HIV/AIDS-related mortality has declined overall since 2010 across Latin American countries, worrisome increases has been reported among women in Costa Rica, El Salvador, Mexico, Panama, Paraguay, and Peru.^6^

In Latin America, a region characterized by marked inequality, the HIV/AIDS epidemic is primarily concentrated in key populations and urban areas, where socioeconomic contrasts can be especially stark.^4^ Studying the influence of living conditions on HIV/AIDS mortality could contribute to the implementation of equity-oriented public policies, especially since there is evidence that social conditions affect HIV/AIDS mortality differently according to age and sex.^7^ As of today, studies on HIV/AIDS-related mortality in Latin American cities remain scarce; however, a multicountry analysis at the municipal level revealed substantial inequalities in mortality due to HIV/AIDS.^5^

To the best of our knowledge, at the time of writing, no previous study had examined the association between HIV/AIDS-related mortality and indicators of living conditions and income inequality across cities in multiple Latin American countries using regionally comparable data. To address this gap, we conducted a study to describe HIV/AIDS-related mortality and its association with indicators of deprivation and income inequality at city-level in Latin American cities with more than 100,000 inhabitants.

## Methods

### Study overview

This is an ecological study using vital registration data from 315 Latin American cities available through the SALURBAL study.^8^ We compiled and harmonized data on health and social indicators from cities that had more than 100,000 inhabitants by 2010 in Argentina, Brazil, Colombia, Costa Rica, Chile, El Salvador, Guatemala, Mexico, Nicaragua, Panama, and Peru.^8^ SALURBAL cities are aggregations of one or more local administrative units (e.g., municipalities) that together form an urban extent determined through geospatial data.^8^ For this study, complete data were available for 315 cities from Argentina, Brazil, Colombia, Chile, El Salvador, Guatemala, Panama, and Mexico.

### Outcome definition and mortality data

To estimate the HIV/AIDS-related mortality rate as health outcome, we used official vital registration mortality data. Within the SALURBAL project, mortality data are harmonized across countries by standardizing variables (age, sex, and cause of death), implementing procedures to address missing data, ill-defined causes, and underreporting thereby ensuring cross-national comparability; further details are provided in reference.^8^ HIV/AIDS was defined as International Classification of Diseases 10th Revision codes B20–B24, as used in the Global Health Estimates study. Mortality data from 2016 to 2020 were analyzed for all countries except Chile, for which the most recent data available in SALURBAL corresponded to 2015–2019. A five-year window was used to account for the relatively low number of deaths per year and to ensure more stable estimates. Data was disaggregated by age and sex (binary). Age was categorized into four groups (14 years old and younger, 15–49 years, 50–69 years, and 70 years and older) to ensure a sufficient number of deaths within each stratum and to reflect age groups with distinct epidemiological and mortality risks in relation to HIV/AIDS. Population denominators data were obtained from national censuses and their corresponding population projections for each city, year, age, and sex.

### Contextual variables

To measure city-level living conditions as the exposure, we used data from the nearest census or national household surveys available for the study period, both harmonized by the SALURBAL study. Deprivation was measured using SEI. Higher SEI values indicate more advantaged conditions, whereas lower values reflect greater structural socioeconomic disadvantage. This index is constructed based on the sum of the z-scores using the mean and standard deviation of the entire distribution of all countries from the SALURBAL study of the following proportion-based indicators: households with access to piped water inside the dwelling, households connected to a public sewage network, households with more than three persons per room (this variable is reverse-coded), and population aged 25 and over who completed primary education or higher.^8^^,^^9^ Each of these variables was used as a general socioeconomic marker and not as a standalone indicator (e.g., of education, overcrowding, access to water, etc.). The SEI was chosen because it has previously demonstrated utility in characterizing population living conditions.^9^

To analyze the association between HIV/AIDS-related mortality and income inequality in each city, we used an income-based Gini coefficient provided by the SALURBAL project, originally calculated by the Comisión Económica para América Latina y el Caribe. This coefficient is a commonly used measure to quantify the inequality in income distribution within a population, taking values from 0 (perfect equality) to 1 (perfect inequality).^10^ We constructed a Gini z-score to align the measurement scale with that of the SEI and to facilitate the interpretation of regression coefficients. In this way, both the SEI and the income-based Gini coefficient are city-specific measures, and both are expressed as z-scores across the distribution of SALURBAL cities. Both contextual variables were harmonized across countries as part of the SALURBAL study, ensuring comparability across different national contexts; further details are provided in reference.^8^

Years availability for income-based Gini coefficient/SEI data was as follows: Argentina 2017/2010, Brazil 2010/2010, Colombia 2017/2005, Chile 2015/2002, El Salvador 2016/2018, Guatemala 2016/2018, Mexico 2010/2020, and Panama 2010/2010.

### Statistical analysis

We conducted this analysis in five steps. First, we described the main characteristics of the cities included in the study by calculating the median and the first and third quartiles (Q1 and Q3) of mortality rates and social indicators.

Second, we estimated age-adjusted mortality rates by city separately for men and women using a hierarchical negative binomial model. The model included age group and country as fixed effects and a city random intercept, with the logarithm of the population included as an offset. From this model, we obtained empirical Bayes predictions of deaths for each city–age group stratum. These model-based age-specific rates were then combined to calculate age-standardized mortality rates (ASMRs) using direct standardization based on the World Health Organization 2000–2025 standard population. No city-level covariates were included in the model; therefore, estimates were shrunk toward the overall mean across cities (see Supplementary Methods 1 for details).

As a separate step we calculated age-specific mortality rates by city for men and women using a similar hierarchical negative binomial models stratified by age group, with country as a fixed effect and city as a random intercept, including the logarithm of the population as an offset. These models were used to obtain empirical Bayes estimates of age-specific mortality rates, providing stabilized estimates in strata with low deaths counts (see Supplementary Methods 2 for details).

Third, to estimate the proportion of variability in HIV/AIDS-related mortality rates attributable to differences between countries and between cities, we used a hierarchical linear model at the city level with the logarithm of the age-adjusted mortality rate as the outcome (as computed from the model above) and a random intercept for country. Variance components obtained from the models were used to estimate intraclass correlation coefficients (ICCs), which assess the proportion of variance explained by differences between countries and between cities (see Supplementary Methods 3 for details).

Fourth, to characterize inequality in the distribution HIV/AIDS-related mortality rates across cities by age and sex, we plotted Lorenz curves and calculated an HIV/AIDS mortality Gini coefficient. For each age-sex group, cities were ordered from lowest to highest HIV/AIDS mortality rate. The Lorenz curve was then generated by plotting the cumulative proportion of deaths against the cumulative proportion of the population. A greater heterogeneity across cities is indicated by a greater deviation of the Lorenz curve from the diagonal line. As a summary measure, we calculate an HIV/AIDS mortality Gini coefficient derived from the corresponding curve. This coefficient quantifies the area between the curve and the line of equality. It ranges from 0 to 1, with values closer to 1 indicating greater inequality in the distribution of mortality rates. This HIV/AIDS mortality Gini coefficient is distinct from the income-based Gini coefficient used as an indicator of income inequality in the regression models. Calculations of HIV/AIDS mortality Gini coefficient and the construction of the Lorenz curve were performed using Epidat version 4·2 (Xunta de Galicia, Galicia, Spain; Pan American Health Organization, Washington D.C., USA; CES University, Colombia) and R version 4·4·3.

Fifth and last, to examine the association between HIV/AIDS mortality and socioeconomic and income inequality characteristics, we started with a bivariate analysis between each variable and the modeled HIV/AIDS age-adjusted mortality rate across all cities. Scatter plots were created to visualize the relationships between these variables, and Spearman's correlation coefficient with 95% confidence intervals estimated via bootstrap with 1000 iterations was calculated. To model sex-stratified associations between HIV/AIDS mortality rates and both the SEI and the income-based Gini coefficient, we fitted four multilevel regression models. We modeled age-by-sex strata nested within cities, incorporating country and age group as fixed effects, city as a random intercept, and the logarithm of the population as an offset. Models 1 and 2 included only the income-based Gini coefficient or the SEI as the main predictors, respectively, whereas Model 3 included both. The fourth and last model incorporated interaction terms between age groups and both the income-based Gini coefficient and the SEI to assess the potential effect modification by age (Model 4 is detailed in Supplementary Methods 4). All models were fitted using the glmmTMB package in R (version 4·4·3).

Because the COVID-19 pandemic may have affected the relationship between the variables analyzed and HIV/AIDS mortality in cities, a sensitivity analysis was conducted. The results were similar to those obtained when including 2020 and are presented in Supplementary Table S1.

All data used in this study were aggregated to ensure confidentiality and to comply with the SALURBAL project's data use agreement. None of the data sources included information that could enable the identification of individuals involved. The SALURBAL study protocol was approved by the Drexel University IRB (ID #1612005035).

### Role of the funding source

The funding source had no role in the study design, data collection, analysis or interpretation of the data, preparation of this manuscript, or the decision to submit the manuscript for publication.

## Results

**Fig. 1:**
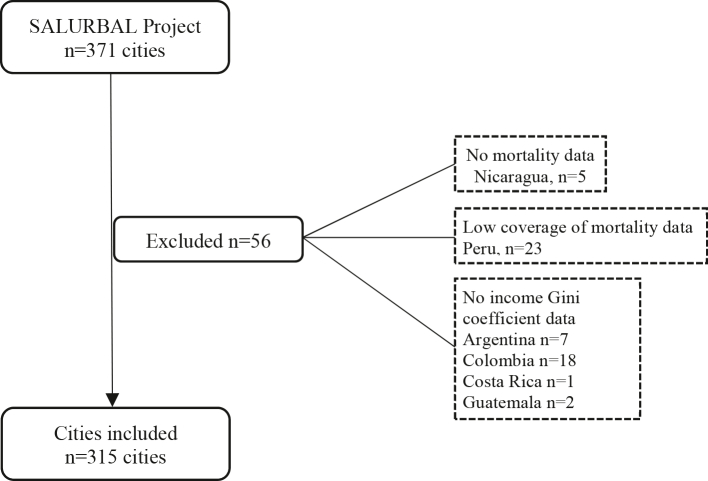
Flow chart of selection and exclusion of the cities included in this study.

A total of 371 cities from 11 Latin American countries were initially available in the SALURBAL database. After excluding cities with mortality data not available, insufficient mortality data coverage, or unavailable income Gini coefficient data, 315 cities (84·9%) were included in the final analysis (Fig. 1). Overall, 56 cities (15·1%) were excluded because of incomplete data.

A total of 78,258 deaths due to HIV/AIDS occurred in the 315 cities studied. The median age-standardized mortality rate (ASMR) for HIV/AIDS was 2·62 per 100,000 women (Q1 = 1·60; Q3 = 4·06) and 7·67 per 100,000 men (Q1 = 5·60; Q3 = 10·30). Overall, the highest ASMRs for HIV/AIDS were observed in cities of Panama, followed by Brazil, Colombia and El Salvador, whereas the lowest ASMRs were found in Argentina, Chile, Guatemala, and Mexico.

Mortality rates were consistently higher among men than women in all countries, and highest in individuals aged 50–69 years, except for Mexican cities, where, on average, HIV/AIDS-related mortality was higher in the 15 to 49-year-old population (Table 1). In terms of living conditions, Chilean cities showed, on average, better conditions (Median SEI = 0·84), whereas those in El Salvador exhibited the worst conditions (Median SEI = −1·15). In terms of income inequality, Brazilian cities had the highest median income inequality (Median Gini = 0·55), whereas Guatemala exhibited the lowest (Table 1).

**Table 1:** Median (Q1; Q3) of the Social Environment Index, income-based Gini coefficient and HIV/AIDS-related mortality rates (per 100,000 inhabitants), by country in 315 Latin American cities.

Country	Argentina	Brazil	Colombia	Chile	Mexico	Guatemala	El Salvador	Panama	Total
Number of cities	26	152	17	21	92	1	3	3	315
Median (Q1; Q3) Income-based Gini coefficient	0·40 (0·37; 0·42)	0·55 (0·52; 0·58)	0·46 (0·44; 0·47)	0·40 (0·39; 0·43)	0·45 (0·42; 0·47)	0·39 –	0·41 (0·39; 0·43)	0·50 (0·46; 0·53)	0·50 (0·43; 0·55)
Median (Q1; Q3) SEI (z-score)	0·38 (0·17; 0·60)	0·12 (−0·25; 0·46)	0·34 (−0·45; 0·57)	0·84 (0·70; 0·92)	0·42 (−0·10; 0·83)	−0·02 –	−1·15 (−1·57; −0·30)	−0·07 (−0·17; 0·43)	0·30 (−0·2; 0·59)
HIV/AIDS-related mortality									
By Sex									
Females									
Number of deaths	16,56	14,005	1303	351	3102	128	208	504	21,257
Median (Q1;Q3) ASMR	1·82 (1·60; 2·36)	3·62 (2·62; 4·77)	3·39 (2·63; 4·21)	1·10 (0·79; 1·41)	1·43 (0·99; 2·30)	1·69	3·56 (2·90; 3·76)	8·37 (7·07; 13·10)	2·62 (1·60; 4·06)
Males									
Number of deaths	3458	29,840	4396	1763	15,399	361	446	1338	57,001
Median (Q1;Q3) ASMR	4·59 (3·89; 6·19)	8·45 (7·16; 10·50)	12·3 (9·31; 13·60)	4·81 (3·85; 6·29)	6·60 (4·72; 10·30)	6·49	10·30 (8·12; 11·10)	20·9 (17·10; 25·60)	7·67 (5·60; 10·30)
By age									
14 years old and younger									
Number of deaths	22	171	11	1	136	12	1	11	365
Median (Q1;Q3) mortality rate	0·08 (0·08; 0·08)	0·12 (0·11; 0·12)	0·08 (0·08; 0·09)	0·01 (0·01; 0·01)	0·15 (0·15; 0·17)	0·28	0·05 (0·04; 0·05)	0·54 (0·51; 0·57)	0·12 (0·11; 0·15)
15–49 years									
Number of deaths	3305	28,950	3843	1347	14,277	330	394	1247	53,693
Median (Q1;Q3) mortality rate	3·91 (3·16; 5·65)	7·18 (5·85; 9·45)	10·90 (7·30; 11·40)	3·97 (2·92; 4·60)	5·63 (3·96; 9·72)	3·50	6·02 (5·45; 6·70)	17·80 (17·0; 25·1)	6·51 (4·56; 9·43)
50–69 years									
Number of deaths	1576	12,968	1599	653	3654	122	184	508	21,264
Median (Q1;Q3) mortality rate	6·13 (5·51; 7·00)	10·40 (9·36; 11·70)	11·3 (9·97; 13·00)	5·07 (4·35; 5·49)	5·37 (4·91; 7·05)	5·95	9·34 (8·15; 9·49)	23·8 (20·10; 29·40)	8·88 (5·83; 10·9)
70 years and older									
Number of deaths	211	1756	246	113	434	25	75	76	2936
Median (Q1;Q3) mortality rate	1·88 (1·59; 2·44)	4·56 (3·64; 5·54)	6·10 (4·86; 7·16)	2·20 (1·82; 2·52)	2·37 (1·93; 3·12)	4·06	10·80 (8·30; 13·00)	9·23 (6·92; 11·40)	3·61 (2·39; 4·95)

Note: Q1 = 1° quartile, Q3 = 3° quartile, SEI = Social Environment Index, ASMR = age-standardized-related mortality rate.

### Variability in HIV/AIDS-related mortality across Latin American cities

**Fig. 2:**
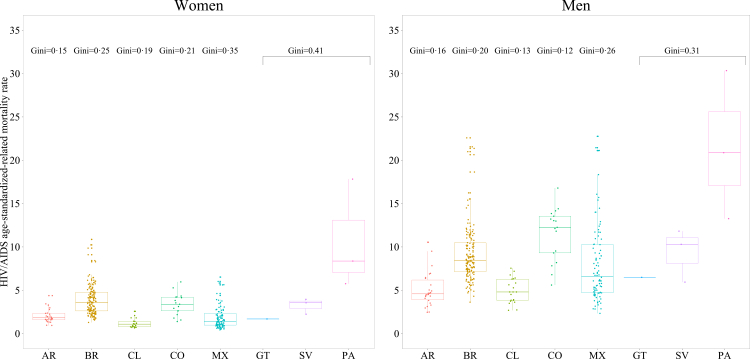
Distribution of HIV/AIDS age-standardized-related mortality rate (per 100,000 inhabitants) by sex in 315 Latin American cities, and Gini coefficient by country. Note: AR = Argentina, BR = Brazil, CL = Chile, CO = Colombia, MX = Mexico, GT = Guatemala, SV = El. Salvador, PA = Panama. The middle line represents the median city HIV/AIDS age-standardized- related mortality rate, the box limits represent the 25th and 75th percentiles, and the whiskers represent 1·5 times the interquartile range. Individual data points (cities) are shown as dots. The HIV/AIDS-related mortality Gini coefficient quantifies the inequality in the distribution of these deaths on a scale from 0 to 1, where 0 indicates perfect equality and 1 indicates maximum inequality.

Fig. 2 shows the distribution of age-adjusted HIV/AIDS-related mortality rates by sex and the Gini coefficient of its distribution across cities in each country. In most countries, the HIV/AIDS-related mortality Gini coefficient was higher for women than for men, indicating a more unequal distribution of deaths, especially in Mexico and Central American countries. The proportion of variance attributable to differences between countries and between cities varied by sex. Among men, 51·7% of the variability in mortality rates was explained by differences between countries, and 48·3% by differences between cities within countries. For women, the variance decomposition showed that 58·5% of the variability was due to differences between countries, while 41·5% was attributable to differences between cities.

**Fig. 3:**
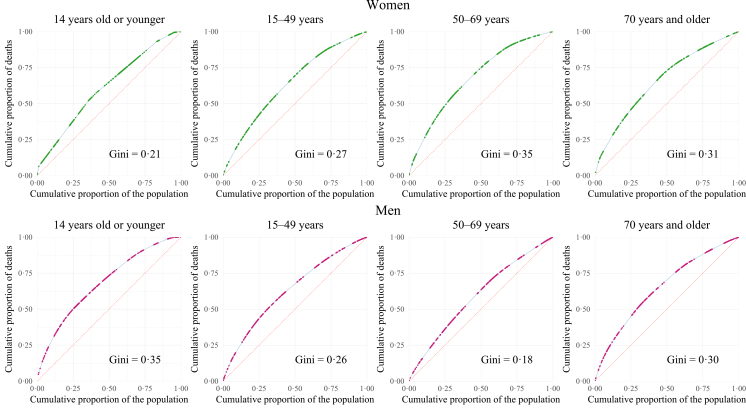
Lorenz curves and HIV/AIDS-related mortality Gini coefficient by sex and age group in 315 Latin American cities. Note: The Lorenz curve shows the cumulative distribution of HIV/AIDS-related deaths as a function of the cumulative distribution of the population. The dashed diagonal line represents perfect equality. The greater the distance between the Lorenz curve and the diagonal, the greater the inequality in the distribution of mortality. The HIV/AIDS-related mortality Gini coefficient quantifies inequality in the distribution of deaths on a scale from 0 to 1, where 0 indicates perfect equality and 1 indicates maximum inequality.

The Lorenz curves and HIV/AIDS-related mortality Gini coefficient by age and sex are shown in Fig. 3. Among women, an increase in inequality in the distribution of deaths was observed with advancing age. The greatest inequality was found in the 50–69 age group (Gini = 0·35). Among men, the highest inequality was observed in the group aged 14 years and under (Gini = 0·35), while the lowest was in the 50–69 age group (Gini = 0·18). When comparing the distributions by age and sex, except for the group aged 14 years and under, all other age categories showed greater inequality in mortality rate distribution among women than among men of the same age, particularly from age 50 onwards (Fig. 3).

### Association between the income-based Gini coefficient, SEI, and HIV/AIDS-related mortality

**Fig. 4:**
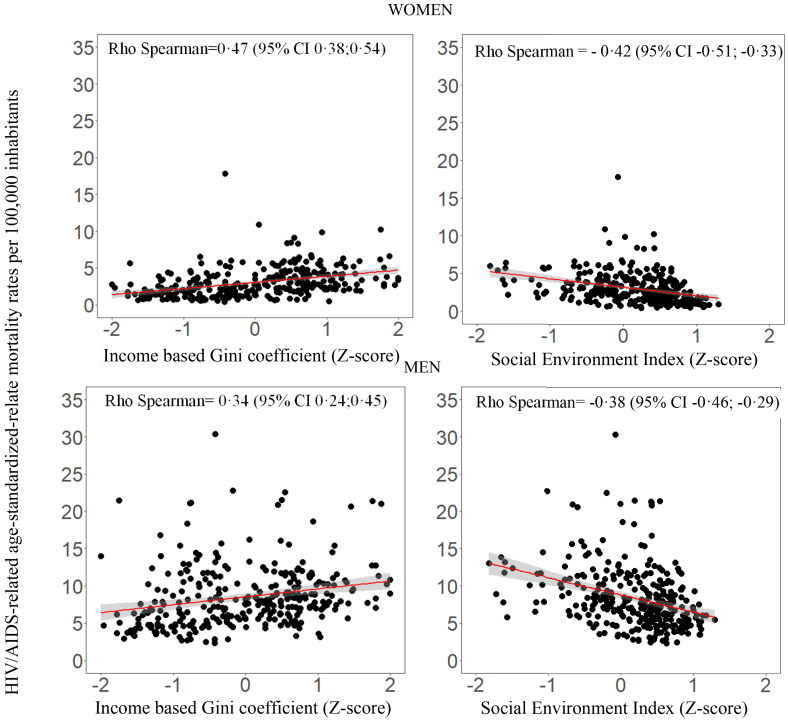
Association between the income-based Gini coefficient and Social Environment Index with HIV/AIDS age-standardized-related mortality rates (per 100,000 inhabitants) by sex in 315 Latin American cities.

The unadjusted associations between HIV/AIDS-related mortality and the income-based Gini coefficient and SEI in Latin American cities are presented in Fig. 4. For both men and women, higher income inequality (i.e., higher Gini coefficient) was associated with higher mortality, while better living conditions (i.e., higher SEI values) were associated with lower mortality. Spearman correlation coefficients ranged from 0·47 to 0·34 for income inequality and from −0·42 to −0·38 for living conditions.

Results of the sex-stratified models are presented in Table 2. Overall, income inequality was not significantly associated with HIV/AIDS-related mortality in both sexes. However, a statistically significant inverse association was observed with SEI. The risk of death from HIV/AIDS was 25% (95% CI 15–30%) lower in women and 19% (95% CI 11–26%) lower in men for each one-point increase in the city-level SEI z-score, after adjusting for country and age (Table 2, Model 3). Mortality rate ratio also varied by age group.

**Table 2:** Association between the income-based Gini coefficient and Social Environment Index with HIV/AIDS-related mortality rate ratios (95% CI) by age and sex in 315 Latin American cities.

	Model 1 RR (95% CI)	Model 2 RR (95% CI)	Model 3 RR (95% CI)
Women			
Income-based Gini coefficient (z-score)	1·00 (0·89; 1·11)		0·98 (0·88; 1·09)
Social Environment Index (z-score)		0·75 (0·67; 0·85)	0·75 (0·70; 0·85)
Ages			
14 years old and younger	0·03 (0·03; 0·04)	0·03 (0·03; 0·04)	0·03 (0·03; 0·04)
15–49 years old	Reference	Reference	Reference
50–69 years old	1·12 (1·06; 1·19)	1·13 (1·06; 1·20)	1·13 (1·06; 1·20)
70 years and older	0·51 (0·46; 0·56)	0·51 (0·47; 0·57)	0·51 (0·47; 0·57)
Men			
Income-based Gini coefficient (z-score)	1·06 (0·98; 1·16)		1·05 (0·97; 1·15)
Social Environment Index (z-score)		0·81 (0·74; 0·89)	0·81 (0·74; 0·89)
Ages			
14 years old and younger	0·01 (0·01; 0·01)	0·01 (0·01; 0·01)	0·01 (0·01; 0·01)
15–49 years old	Reference	Reference	Reference
50–69 years old	1·26 (1·20; 1·31)	1·26 (1·20; 1·32)	1·26 (1·20; 1·32)
70 years and older	0·63 (0·59; 0·68)	0·63 (0·59; 0·68)	0·63 (0·59; 0·68)

**Note**: RR = rate ratio, CI = confidence interval. Model 1 includes age group and the income-based Gini coefficient; Model 2 includes age group and Social Environment Index; and Model 3 includes age group, the income-based Gini coefficient, and Social Environment Index. All models were adjusted for country (fixed effect) and city (random effect). The income-based Gini coefficient and Social Environment Index are standardized as z-scores.

The results of the full model are presented in Supplementary Table S2. This model included interactions between both socioeconomic variables and age. The lack of association with income inequality remained, while the inverse association with SEI persisted. This association was observed across all age groups, except among women aged 70 years and older. In the population aged 14 years and under, the strongest association with SEI was observed and the risk of death was 43% lower in women and 50% lower in men for each z-score unit increase in SEI.

## Discussion

Our findings show that HIV/AIDS mortality rates were markedly unequal across countries and cities included in this study, with mortality rates varying up to 10-fold between cities. Age-specific mortality rates were higher among men than women, although the distribution was more unequal among women. An important part of the variability was explained by differences between cities. We also found that cities with better socioeconomic conditions exhibited lower HIV/AIDS mortality in both women and men, with these associations being especially strong for the youngest age groups. While we found some associations between HIV/AIDS mortality and income inequality, this association was not robust to age adjustment.

Higher mortality among men aligns with global epidemiological patterns. Given the ecological design of our study, these findings should not be interpreted as reflecting individual-level mechanisms. Rather, this pattern may be understood as being consistent with the broader population distribution of HIV in the region, including the historically higher prevalence among men who have sex with men, as well as contextual barriers to timely access to healthcare services.^11^

Among women, the Lorenz curves revealed greater inequality in the distribution of deaths among those aged 50 and older. This may be related to population-level gaps in the orientation of HIV/AIDS prevention and control strategies for women, which have predominantly focused on maternal and child health, while paying less attention to non-reproductive-related contexts.^12^ Moreover, this population may face specific forms of social and institutional stigmatization, as well as barriers linked to gender stereotypes, which may hinder access to information and health services. For instance, prejudices and misconceptions that assume older women are not sexually active may delay HIV testing and thereby contribute to underdiagnosis.^12^ These challenges, combined with rising HIV prevalence among older adults,^13^ may be implicated in the mortality inequalities observed in this population. This emerging demographic shift warrants focused investigation across Latin American contexts. In addition to this, lower mortality rates on younger women might be observed due lower HIV diagnosis rates given that non-pregnant women are not necessarily a target of HIV/AIDS diagnosis and treatment programs.^12^ Thus, deaths in this population might be occurring but being reported only as opportunistic infections.^14^

An important proportion of the variability in HIV/AIDS mortality rates was explained by between-country differences, accounting for approximately 51·6% among males and 58·5% among females, reflecting persistent structural and policy determinants. Despite strong national autonomy in resource allocation and the widespread implementation of free HIV/AIDS diagnosis and treatment programs since the late 1990s, outcomes remain suboptimal, and many countries have not yet achieved the 90-90-90 targets.^6^^,^^15^ Our findings suggest that while national autonomy and expanded treatment access represent critical steps forward, there remains considerable opportunity to enhance the effectiveness and equity of HIV/AIDS responses through structural and policy efforts.

Differences at city level within countries also accounted for a large share of the variability (48·3% in men and 41·5% in women) highlighting the importance of urban-level determinants in shaping health inequalities. This finding is particularly relevant, as it underscores the importance of the city as a meaningful scale for understanding HIV/AIDS mortality. For example, previous reports have shown that about 15 cities in Brazil account for 60% of people living with HIV at the national level.^16^ In Latin America, where urbanization is intense and socially heterogeneous, national averages can mask substantial differences between cities within the same country. In this sense, our results suggest that efforts to reduce HIV/AIDS mortality may require not only sustained national policies, but also interventions that respond to local urban contexts. Although urban areas are home to the majority of people living with HIV/AIDS, few studies have examined HIV/AIDS mortality patterns across multiple cities. Although not directly comparable, our results are convergent with reports describing wide inequalities between cities.^5^

We also studied the association of HIV/AIDS mortality with income inequality and living conditions. Our bivariate analysis showed positive and moderate association between higher income-based Gini coefficient and higher HIV/AIDS mortality in both women and men; however, this association did not persist after age-adjustment and between country differences. This lack of association after adjustment for country may reflect that city-level income inequality exhibits limited variability within countries, compared with the larger differences observed between countries, thereby reducing its explanatory power once the national context is taken into account. Previous research has shown that the relationship between the income-based Gini coefficient and various health outcomes tends to vary depending on the outcomes, methods, and analytical units used.^17^ This association is usually more consistent when comparing large areas such as countries, and less consistent at subnational or small-area levels. This may be explained by the way national-level inequality shapes the overall degree of social stratification, which in turn influences the pattern observed in more disaggregated levels.^17^ The lack of association observed after adjusting the model does not necessarily imply that inequality in income distribution is irrelevant. Rather, its effects may operate through processes at another level that are not fully captured by our analysis.

Our analysis did reveal that cities with more disadvantaged living conditions exhibited significantly higher HIV/AIDS mortality rates, suggesting socioeconomic determinants play an important role in urban health outcomes. This finding complements previous research that examined this association within cities. In European cities, higher HIV/AIDS mortality rates have been documented in neighborhoods with unfavorable socioeconomic indicators, such as lower education levels, higher unemployment, or material deprivation.^18^, ^19^, ^20^

Within Latin America, existing research on HIV/AIDS mortality disparities has been largely confined to Brazilian urban contexts. Studies in Brasília demonstrate a direct association between low-income neighborhoods and higher HIV/AIDS mortality,^21^ while findings from São Paulo, present inconsistent results regarding this relationship.^22^, ^23^, ^24^ Although the underlying processes driving these inequalities remain incompletely understood and are beyond the scope of our study, previous publications have interpreted them in light of social and contextual factors, including the higher prevalence of intravenous drug use in certain urban areas, poverty, gender-related discrimination, and other forms of social marginalization. These factors, among others, may shape the broader contexts in which HIV care processes take place and could contribute to inequalities in mortality across cities.^19^^,^^22^^,^^25^ These effects have been examined mostly in studies focusing separately on individual or area-level factors; however, one multilevel study demonstrated an independent effect on HIV/AIDS mortality at the community level.^19^

This study has some limitations that should be considered. First, on our city-level conditions, information on income inequality and living conditions was available for different years not necessarily aligned with mortality data. Limited availability of socioeconomic data has historically constrained public health surveillance databases,^26^ and in our case this challenge is further compounded by the need for measures that are comparable across countries and cities. Although the temporal mismatch between the social indicators and mortality data is a limitation, previous studies have used census-based measures from a single point in time to characterize area-level socioeconomic conditions^27^ including in prior work where we examined the impact of this misalignment and found it to not affect inferences substantially^28^ We believe that the use of these indicators is justified because they capture relatively stable structural characteristics of places, particularly when the geographic units are larger than census tracts.^26^^,^^29^ In our study, these indicators were used to establish a gradient of social conditions across cities. These considerations should be interpreted in light of both the limited availability of updated data and the descriptive scope of our analyses, which were intended to assess associations rather than causality. The impact of this temporal mismatch may have been limited if the relative socioeconomic position of cities remained stable over time.^28^^,^^30^

Second, on our outcome, we relied on vital registration to quantify HIV/AIDS-related deaths. Although this source enables comparability across cities and countries, it may be influenced by differences in the quality of cause of death certification and the coverage of registration systems. It is important to note that, within the SALURBAL project, mortality data are harmonized across countries by standardizing variables (age, sex, and cause of death), implementing procedures to address missing data, ill-defined causes, and undercounting of mortality thereby ensuring cross-national comparability; further details are provided in reference.^8^

Third, one of the main limitations of this study is our limited ability to include other relevant variables related to HIV/AIDS mortality, such as HIV prevalence, late diagnosis, antiretroviral therapy coverage, testing coverage, quality of healthcare, and other contextual factors. This was mainly due to the lack of available data at the city level across countries. As a result, our models may not fully capture important dimensions of inequality in HIV/AIDS-related mortality, such as the HIV care cascade and the associations observed should be interpreted considering other contextual factors operating at multiple levels.

Fourth, although a five-year period and appropriate statistical methods were used to smooth estimates, mortality rates in women over 50 years old may be affected by instability in areas with few deaths and thus should be interpreted with caution.

A further limitation of this study is that findings may not be generalizable to all cities in Latin America, as the cities included as unit of analysis had 100,000 or more inhabitants across 8 of the 11 countries participating in the SALURBAL project. Results should therefore be interpreted with caution, given that smaller cities and countries not represented in this study may exhibit different patterns.

Despite these limitations, the data used in this study represents the best available evidence and provides a valuable starting point for developing an urban-level perspective on inequalities in HIV/AIDS mortality across major cities in Latin America. To our knowledge, at the time this manuscript was written, no previous study had examined inter-urban inequalities in HIV/AIDS mortality across multiple Latin American countries in relation to social determinants, stratified by age and sex. The contribution of this study lies not simply in documenting that socially disadvantaged urban environments experience higher HIV/AIDS mortality, but in showing how these patterns may reflect missed opportunities even within the same country. At the same time, by enabling comparisons across cities in multiple Latin American countries, the study helps to identify feasible targets for epidemic control. Overall, the findings underscore the relevance of the city as an epidemiologically meaningful scale at which structural inequalities may shape mortality patterns, even in contexts where national public policies, in theory, guarantee access to care and follow-up. This study also highlights critical gaps for future research while providing an evidence base to inform more tailored public health strategies and equity-oriented policies aimed at reducing avoidable inequalities in HIV/AIDS mortality.

## Contributors

SG, and AT conceived the study.

SG did the statistical analyses and visualization.

SG drafted the first version of the manuscript.

SG, UB, AT, MSP and MA directly accessed and verified the underlying data reported in the manuscript.

TA, DHM, LM participated in or supported data collection.

All authors participated in writing, review and editing the manuscript.

All authors participated in the interpretation of results and approved the final version of the manuscript.

## Data sharing statement

The SALURBAL project welcomes queries from anyone interested in learning more about its dataset and potential access to data. To learn more about SALURBAL's dataset, visit the SALURBAL project website or contact the project at salurbal@drexel.edu. After publication of this study, study protocols, data dictionaries, and requested study data may be made available to interested investigators after they have signed a data use agreement with SALURBAL and if their study proposal, developed in collaboration with SALURBAL investigators, is approved by the SALURBAL proposal and publications committee. Some data may not be available to external investigators because of data confidentiality agreements.

## Declaration of generative AI and AI-assisted technologies in the manuscript preparation process

During the preparation of this work Sebastián Genero used ChatGPT (OpenAI) to assist with language editing and to enhance the clarity of the manuscript. No full drafts or analytical decisions were based on or performed by any artificial intelligence tool. All analyses were conducted independently by the authors. After using this tool, the authors reviewed and edited the content as needed and take full responsibility for the content of the published article.

## Declaration of interests

AT reports institutional grants from Wellcome Trust and the Medical Research Council. MSP reports payments from Drexel University under a Wellcome Trust grant. DHM reports an institutional grant from Wellcome Trust and a PhD stipend from Fulbright/Minciencias. MA reports an institutional grant from Wellcome Trust. TA reports payments from Drexel University under a Wellcome Trust grant; payments from CECAN (Fondo de Financiamiento de Centros de Investigación en Áreas Prioritarias, FONDAP, Government of Chile); consultancy fees from Organon for the development of a list of women's health indicators; and travel support from Drexel University under the Wellcome Trust grant to attend SALURBAL meetings. UB reports institutional grants from the National Institutes of Health and Wellcome Trust. SG and LM declare no competing interests.
