# Supplementary material for: Inequities in HIV/AIDS mortality in 315 Latin American cities: a population-based cross-sectional analysis
Source: Lancet Reg Health Am. 2026 Jul 20;62:101580. doi: 10.1016/j.lana.2026.101580 (PMC13393700; doi:10.1016/j.lana.2026.101580)
Supplement: Supplementary Methods and Tables [file mmc1.pdf]

Inequities in HIV/AIDS mortality in 315 Latin American cities: a population-based cross-sectional analysis

Table of contents

Supplementary Methods 1 2

Supplementary Methods 2 3

Supplementary Methods 3 4

Supplementary Methods 4 5

Supplementary Table 1 6

Supplementary Table 2 7

Supplementary Methods 1

To estimate age-adjusted mortality rates by city for men and women separately, we fitted a negative binomial multilevel model:

$$Y_{ijk} \sim \text{NegBin}(\mu_{ijk,}\ \theta)$$

$$\log(\mu_{ijk}) = \log(N_{ijk}) + \beta_0 + \beta_1\ \text{AgeGroup}_i + \beta_2\ \text{Country}_k + u_j$$

$$u_j \sim N\ (0, \sigma^2)$$

where Y_{ijk} represents the number of deaths in age group *i*, city *j* nested within country *k*, NegBin denotes the negative binomial distribution with mean μ_{ijk} and dispersion parameter θ; N_{ijk} is the corresponding population and was included as an offset; β_0 is the model intercept; β_1 and β_2 are fixed-effect coefficients for age group and country, respectively. The term u_j is a random intercept for city and N $(0, \sigma^2)$ denotes the normal distribution with mean 0 and variance σ^2. AgeGroup denotes categorical age groups.

From this model, we obtained empirical Bayes predictions of deaths for each city-age group stratum, which we used to calculate age-specific rates. These model-based age-specific rates were then combined into an age-standardized mortality rate using direct standardization based on the World Health Organization 2000–2025 standard population.

Supplementary Methods 2

To estimate age-specific mortality rates by city for men and women separately, we fitted a negative binomial multilevel model for each age group (0-14, 15-49, 50-69, and 70 and older):

$$Y_{ij} \sim \text{NegBin}(\mu_{ij}, \theta)$$

$$\log(\mu_{ij}) = \log(N_{ij}) + \beta_0 + \beta_1 \text{ Country}_j + u_i$$

$$u_i \sim N(0, \sigma^2)$$

where Y_{ij} represents the number of deaths in city *i* nested within country *j*, NegBin denotes the negative binomial distribution with mean μijk and dispersion parameter θ; N_{ij} is the corresponding population, included as an offset. β_0 is the model intercept; β_1 is the fixed coefficient for country. The term u_i is a random intercept for city and N (0, σ^2) denotes the normal distribution with mean 0 and variance σ^2. From this model, we obtained empirical Bayes predictions of deaths for each city, age group, and sex.

Supplementary Methods 3

To partition variance in between and within country variability, we fitted separate linear multilevel models by sex:

$$Y_{ij} = \beta_0 + u_j + \varepsilon_{ij}$$

$$u_j \sim N(0, \tau_{00})$$

$$\varepsilon_{ij} \sim N(0, \sigma^2)$$

$$ICC = \tau_{00} / (\tau_{00} + \sigma^2)$$

where Y_{ij} is the age-adjusted mortality rate by city *i* nested within country j (estimated as described in Supplementary Methods 1), β_0 is the intercept; u_j is a random intercept for country (with variance τ_{00}) and ε_{ij} is the city residual. The intraclass correlation coefficient is computed as τ_{00} divided by the total variance.

Supplementary Methods 4

To study the association between the income-based Gini coefficient, Social Environment Index, and HIV-AIDS-related mortality, we fitted negative binomial multilevel models by sex:

$$Y_{ijk} \sim NegBin(\mu_{ijk}, \theta)$$

$$\log(\mu_{ijk}) = \log(N_{ijk}) + \beta_0 + \beta_1 \times AgeGroup_{ijk} + \beta_2 Gini_{jk} + \beta_3 SEI_{jk} +$$

$$\beta_4(AgeGroup_{ijk} \times Gini_{jk}) + \beta_5(AgeGroup_{ijk} \times SEI_{jk}) +$$

$$\beta_6 Country_k + u_j$$

$$u_j \sim N(0, \sigma^2)$$

where Y_{ijk} represents the number of deaths in age group *i*, city *j* nested within country *k;* NegBin denotes the negative binomial distribution with mean μ_{ijk} and dispersion parameter θ; N_{ijk} is the corresponding population included as an offset. The term u_j is a random intercept for city. B_0 is the intercept, β_1, β_2, and β_3 are the coefficients associated with age group, income-based Gini coefficient, and SEI respectively; β_4 and β_5 represent the interaction terms of income-based Gini coefficient and SEI with age group, respectively; and β_6 is the fixed effect for country. AgeGroup denotes categorical age groups; Gini denotes the income-based Gini coefficient and SEI denotes the Social Environment Index.

Supplementary Table 1:Results of the sensibility analysis: associations between the income-based Gini coefficient and Social Environment Index with HIV/AIDS-related mortality rate ratios (95% CI) by age and sex in 315 Latin American cities, 2016-2019.

	Model 1<br>RR (95% CI)	Model 2<br>RR (95% CI)	Model 3<br>RR (95% CI)
Women			
Income-based Gini coefficient (z-score)	0·98 (0·88; 1·10)		0·97 (0·87;1·08)
Social Environment Index (z-score)		0·74 (0·66;0·85)	0·74 (0·65;0·83)
Ages			
14 years old and younger	0·04 (0·03; 0·04)	0·04 (0·03; 0·04)	0·04 (0·03; 0·04)
15-49 years old	Reference	Reference	Reference
50-69 years old	1·10 (1·03;1·17)	1·10 (1·03;1·18)	1·10 (1·03;1·18)
70 years and older	0·48 (0·43;0·53)	0·48 (0·43;0·53)	0·48 (0·43;0·53)
Men			
Income-based Gini coefficient (z-score)	1·06 (0·97;1·15)		1·05 (0·96;1·14)
Social Environment Index (z-score)		0·81 (0·74;0·89)	0·81 (0·74; 0·89)
Ages			
14 years old and younger	0·01 (0·01;0·01)	0·01 (0·01;0·01)	0·01 (0·01;0·01)
15-49 years old	Reference	Reference	Reference
50-69 years old	1·26 (1·20;1·31)	1·26 (1·20;1·32)	1·26 (1·20;1·32)
70 years and older	0·62 (0·58;0·67)	0·62 (0·58;0·67)	0·62 (0·58;0·67)

Note: Model 1 includes age group and the income-based Gini coefficient; Model 2 includes age group and Social Environment Index; and Model 3 includes age group, the income-based Gini coefficient, and Social Environment Index. All models were adjusted for country (fixed effect) and city (random effect). The income-based Gini coefficient and Social Environment Index are standardized as z-scores.

Supplementary Table 2: Association between the income-based Gini coefficient and Social Environment Index with HIV/AIDS-related mortality rate ratios (95% CI) by sex and age group in 315 Latin American cities (Model 4)

	Women	Men
	RR (CI 95%)	RR (CI 95%)
Income-based Gini coefficient (z-score)		
14 years old and younger	0.85 (0.71;1.01)	1.05 (0.90;1.23)
15-49 years old	0.98 (0.87;1.09)	1.03 (0.94;1.12)
50-69 years old	1.00 (0,89;1,13)	1.09 (1.00;1.19)
70 years and older	0.97 (0.85;1.11)	1.09 (0.98;1.2)
Social Environment Index (z-score)		
14 years old and younger	0.57 (0.42;0.77)	0.50 (0.38;0.65)
15-49 years old	0.76 (0.67;0.87)	0.83 (0.76;0.92)
50-69 years old	0.74 (0.64;0.85)	0.85 (0.76;0.94)
70 years and older	0.83 (0.67;1.03)	0.63 (0.55;0.73)

Note: Results correspond to exponentiated interaction terms from model 4, which includes age group, the income-based Gini coefficient, SEI, and their interaction terms (income-based Gini coefficient × age group and Social Environment Index × age group). Each coefficient shown in this table represents the linear combination of the Gini (or Social Environment Index) and the age interaction terms, so they can be interpreted as the effect of Gini (or Social Environment Index) in each age group separately. All models were adjusted for country (fixed effect) and city (random effect). The income-based Gini coefficient and Social Environment Index are standardized as z-scores.
